# Supplementary material for: Unveiling the Bioactive Potential of the Invasive Jellyfish Phyllorhiza punctata Through Integrative Transcriptomic and Proteomic Analyses
Source: Biomolecules. 2025 Aug 4;15(8):1121. doi: 10.3390/biom15081121 (PMC12383608; doi:10.3390/biom15081121)
Supplement: Supplementary file 1 [file biomolecules-15-01121-s001.zip › Suplementary Material/FileS3/PEPFOLD4/XP_065052735_11090/clusters.html]

 PEP-FOLD clusters


Download archive of all models  here   

|  |  |  |
| --- | --- | --- |
| # | file | sOPEP |
| # cluster 1 - Size: 7 |  |  |
|  | XP\_065052735\_11090-model1 | -11.155 |
|  | XP\_065052735\_11090-model1.1 | -11.155 |
|  | XP\_065052735\_11090-model1.2 | -11.155 |
|  | XP\_065052735\_11090-model1.3 | -11.155 |
|  | XP\_065052735\_11090-model1.4 | -11.155 |
|  | XP\_065052735\_11090-model1.5 | -11.155 |
|  | XP\_065052735\_11090-model1.6 | -11.0932 |
| # cluster 2 - Size: 18 |  |  |
|  | XP\_065052735\_11090-model2 | -7.35616 |
|  | XP\_065052735\_11090-model2.1 | -7.1579 |
|  | XP\_065052735\_11090-model2.2 | -6.92561 |
|  | XP\_065052735\_11090-model2.3 | -6.87046 |
|  | XP\_065052735\_11090-model2.4 | -6.79016 |
|  | XP\_065052735\_11090-model2.5 | -6.63042 |
|  | XP\_065052735\_11090-model2.6 | -6.61552 |
|  | XP\_065052735\_11090-model2.7 | -6.6023 |
|  | XP\_065052735\_11090-model2.8 | -6.6023 |
|  | XP\_065052735\_11090-model2.9 | -6.50672 |
|  | XP\_065052735\_11090-model2.10 | -6.44719 |
|  | XP\_065052735\_11090-model2.11 | -6.44021 |
|  | XP\_065052735\_11090-model2.12 | -6.2408 |
|  | XP\_065052735\_11090-model2.13 | -6.2176 |
|  | XP\_065052735\_11090-model2.14 | -6.2176 |
|  | XP\_065052735\_11090-model2.15 | -5.91183 |
|  | XP\_065052735\_11090-model2.16 | -5.8504 |
|  | XP\_065052735\_11090-model2.17 | -5.68901 |
| # cluster 3 - Size: 17 |  |  |
|  | XP\_065052735\_11090-model3 | -7.10073 |
|  | XP\_065052735\_11090-model3.1 | -6.88228 |
|  | XP\_065052735\_11090-model3.2 | -6.82642 |
|  | XP\_065052735\_11090-model3.3 | -6.82642 |
|  | XP\_065052735\_11090-model3.4 | -6.78531 |
|  | XP\_065052735\_11090-model3.5 | -6.73278 |
|  | XP\_065052735\_11090-model3.6 | -6.64075 |
|  | XP\_065052735\_11090-model3.7 | -6.62572 |
|  | XP\_065052735\_11090-model3.8 | -6.62572 |
|  | XP\_065052735\_11090-model3.9 | -6.47755 |
|  | XP\_065052735\_11090-model3.10 | -6.33973 |
|  | XP\_065052735\_11090-model3.11 | -6.29529 |
|  | XP\_065052735\_11090-model3.12 | -6.19416 |
|  | XP\_065052735\_11090-model3.13 | -5.96049 |
|  | XP\_065052735\_11090-model3.14 | -5.86724 |
|  | XP\_065052735\_11090-model3.15 | -5.73914 |
|  | XP\_065052735\_11090-model3.16 | -5.69165 |
| # cluster 4 - Size: 4 |  |  |
|  | XP\_065052735\_11090-model4 | -6.80283 |
|  | XP\_065052735\_11090-model4.1 | -6.44928 |
|  | XP\_065052735\_11090-model4.2 | -6.28587 |
|  | XP\_065052735\_11090-model4.3 | -6.27406 |
| # cluster 5 - Size: 7 |  |  |
|  | XP\_065052735\_11090-model5 | -6.7578 |
|  | XP\_065052735\_11090-model5.1 | -6.7578 |
|  | XP\_065052735\_11090-model5.2 | -6.64542 |
|  | XP\_065052735\_11090-model5.3 | -6.59532 |
|  | XP\_065052735\_11090-model5.4 | -6.51175 |
|  | XP\_065052735\_11090-model5.5 | -6.29931 |
|  | XP\_065052735\_11090-model5.6 | -6.26072 |
| # cluster 6 - Size: 28 |  |  |
|  | XP\_065052735\_11090-model6 | -6.72914 |
|  | XP\_065052735\_11090-model6.1 | -6.68019 |
|  | XP\_065052735\_11090-model6.2 | -6.67664 |
|  | XP\_065052735\_11090-model6.3 | -6.6764 |
|  | XP\_065052735\_11090-model6.4 | -6.63408 |
|  | XP\_065052735\_11090-model6.5 | -6.63088 |
|  | XP\_065052735\_11090-model6.6 | -6.6304 |
|  | XP\_065052735\_11090-model6.7 | -6.60353 |
|  | XP\_065052735\_11090-model6.8 | -6.59462 |
|  | XP\_065052735\_11090-model6.9 | -6.58588 |
|  | XP\_065052735\_11090-model6.10 | -6.55467 |
|  | XP\_065052735\_11090-model6.11 | -6.53498 |
|  | XP\_065052735\_11090-model6.12 | -6.4793 |
|  | XP\_065052735\_11090-model6.13 | -6.47357 |
|  | XP\_065052735\_11090-model6.14 | -6.39544 |
|  | XP\_065052735\_11090-model6.15 | -6.34194 |
|  | XP\_065052735\_11090-model6.16 | -6.28138 |
|  | XP\_065052735\_11090-model6.17 | -6.27197 |
|  | XP\_065052735\_11090-model6.18 | -6.23952 |
|  | XP\_065052735\_11090-model6.19 | -6.23447 |
|  | XP\_065052735\_11090-model6.20 | -6.22531 |
|  | XP\_065052735\_11090-model6.21 | -6.15734 |
|  | XP\_065052735\_11090-model6.22 | -6.14302 |
|  | XP\_065052735\_11090-model6.23 | -6.11141 |
|  | XP\_065052735\_11090-model6.24 | -6.05289 |
|  | XP\_065052735\_11090-model6.25 | -6.05034 |
|  | XP\_065052735\_11090-model6.26 | -5.9467 |
|  | XP\_065052735\_11090-model6.27 | -5.91199 |
| # cluster 7 - Size: 12 |  |  |
|  | XP\_065052735\_11090-model7 | -6.72387 |
|  | XP\_065052735\_11090-model7.1 | -6.72387 |
|  | XP\_065052735\_11090-model7.2 | -6.70211 |
|  | XP\_065052735\_11090-model7.3 | -6.53014 |
|  | XP\_065052735\_11090-model7.4 | -6.48573 |
|  | XP\_065052735\_11090-model7.5 | -6.47734 |
|  | XP\_065052735\_11090-model7.6 | -6.36826 |
|  | XP\_065052735\_11090-model7.7 | -6.32186 |
|  | XP\_065052735\_11090-model7.8 | -6.31907 |
|  | XP\_065052735\_11090-model7.9 | -6.21251 |
|  | XP\_065052735\_11090-model7.10 | -6.1557 |
|  | XP\_065052735\_11090-model7.11 | -5.80119 |
| # cluster 8 - Size: 7 |  |  |
|  | XP\_065052735\_11090-model8 | -6.56513 |
|  | XP\_065052735\_11090-model8.1 | -6.56513 |
|  | XP\_065052735\_11090-model8.2 | -6.27961 |
|  | XP\_065052735\_11090-model8.3 | -6.11984 |
|  | XP\_065052735\_11090-model8.4 | -5.92327 |
|  | XP\_065052735\_11090-model8.5 | -5.83642 |
|  | XP\_065052735\_11090-model8.6 | -5.7621 |
|  |  |  |
